# Crystal structure of *catena*-poly[[silver(I)-*μ*-*N*-(pyridin-2-ylmeth­yl)pyridine-3-amine-κ^2^ *N*:*N*′] tri­fluoro­methane­sulfonate]

**DOI:** 10.1107/S1600536814022922

**Published:** 2014-10-24

**Authors:** Suk-Hee Moon, Seonghwa Cho, Ki-Min Park

**Affiliations:** a Department of Food & Nutrition, Kyungnam College of Information and Technology, Busan 617-701, Republic of Korea; b Department of Chemistry, Gyeongsang National University, Jinju 660-701, Republic of Korea; c Research Institute of Natural Sciences, Gyeongsang National University, Jinju 660-701, Republic of Korea

**Keywords:** crystal structure, silver(I), unsymmetrical dipyridyl ligand, helical chain coordination polymer

## Abstract

In the asymmetric unit of the title compound, {[Ag(C_11_H_11_N_3_)]CF_3_SO_3_}_*n*_, there are two Ag^I^ atoms, two *N*-(pyridine-2-ylmeth­yl)pyridine-3-amine ligands (*A* and *B*) and two CF_3_SO_3_^−^ anions. Both Ag^I^ atoms are bridged by two pyridine N atoms from two symmetry-related *A* or *B* ligands, forming right- or left-handed helical chains, respectively. The Ag^I^ atom of the right-handed helical chain adopts a slightly distorted linear coordination geometry [N—Ag—N = 170.69 (14)°], while that of the left-handed helical chain adopts a bent geometry [N—Ag—N = 149.42 (14)°]. Both helical chains have the same pitch length [10.8437 (5) Å], propagate along the *b*-axial direction and are alternately arranged *via* Ag⋯Ag [3.0814 (5) Å] and π–π stacking inter­actions [centroid–centroid distances = 3.514 (3) and 3.487 (3) Å], resulting in the formation of a two-dimensional supra­molecular network extending parallel to the *ab* plane. Weak Ag⋯O [2.861 (4), 2.617 (3), and 2.624 (4) Å] and Ag⋯F [3.017 (3) Å] inter­actions as well as N—H⋯O and C—H⋯O, C—H⋯N and C—H⋯F hydrogen-bonding inter­actions occur between the helical chains and the anions.

## Abstract 2

The reaction of Ag^I^ with the unsymmetrical ligand *N*-(pyridine-2-ylmeth­yl)pyridine-3-amine afforded right- and left-handed helical chains. The Ag^I^ atom of the right-handed helical chain adopts a slightly distorted linear coordination geometry, while that of the left-handed helical chain displays a bent geometry.

## Chemical context   

A few silver coordination polymers based on unsymmetrical dipyridyl ligands composed of two terminal pyridines with different substituted-nitro­gen positions have been reported (Moon & Park, 2013 ▶, 2014 ▶; Zhang *et al.*, 2013 ▶). In an extension of investigations on Ag^I^ coordination polymers with unsymmetrical dipyridyl ligands, the title compound was prepared by the reaction of silver tri­fluoro­metane­sulfonate with *N*-(pyridine-2-ylmeth­yl)pyridine-3-amine. The structure of title compound is related to that of the perchlorate salt (Moon & Park, 2014 ▶; Zhang *et al.*, 2013 ▶).
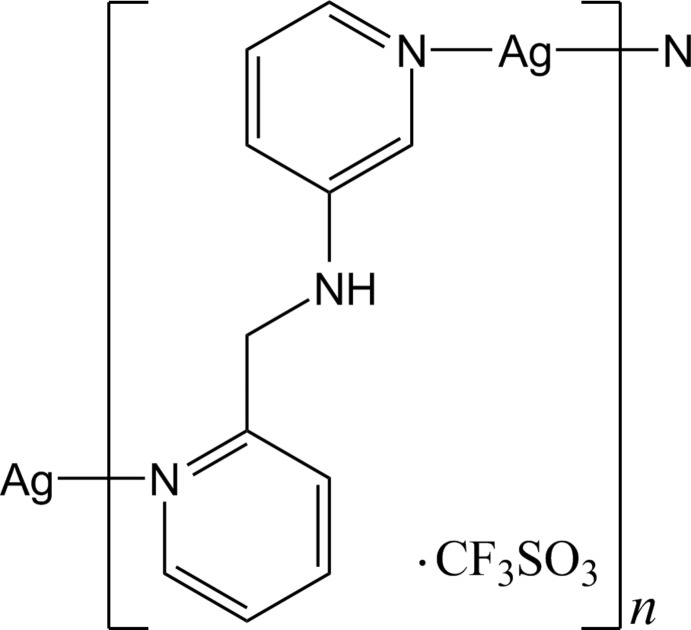


## Structural commentary   

**Figure 1:**
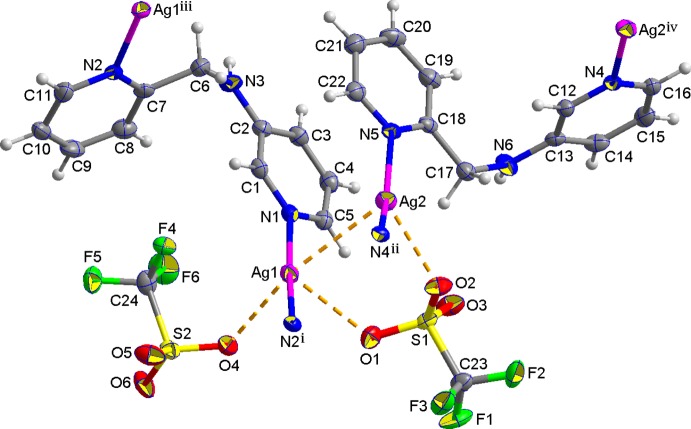
A view of the mol­ecular structure of the title compound, with the atom numbering. Displacement ellipsoids are drawn at the 50% probability level and dashed lines represent Ag⋯Ag and Ag⋯O inter­actions [symmetry codes: (i) −*x* + 1, *y* + 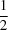, −*z* + 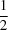; (ii) −*x*, *y* + 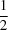, −*z* + 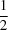; (iii) −*x* + 1, *y* − 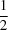, −*z* + 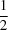; (iv) −*x*, *y* − 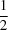, −*z* + 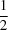].

The molecular components of the title structure are shown in Fig. 1 ▶. The asymmetric unit contains two Ag^I^ atoms (Ag1 and Ag2), two *N*-(pyridine-2-ylmeth­yl)pyridine-3-amine (Lee *et al.*, 2013 ▶) ligands (*A* and *B*) and two tri­fluoro­methane­sulfonate anions. The Ag1 atom is coordinated by two pyridine N atoms from two symmetry-related *A* ligands giving a geometry which is slightly distorted from linear [N1—Ag1—N2 = 170.69 (14)°], forming a right-handed helical chain, while the Ag2 atom is coordinated by two pyridine N atoms from two symmetry-related *B* ligands in a bent arrangement [N4—Ag2—N5 = 149.42 (14)°], forming a left-handed helical chain. Two pyridine rings coordinating to the Ag1 and Ag2 atoms are tilted by 14.1 (3) and 28.9 (2)°, respectively, with respect to each other.

## Supra­molecular features   

**Figure 2:**
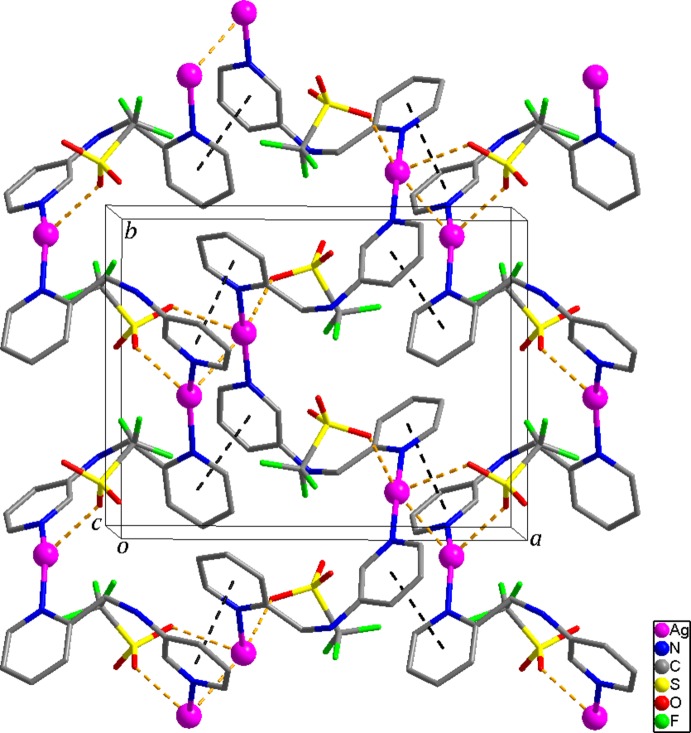
The two-dimensional supra­molecular network formed through Ag⋯Ag and Ag⋯O inter­actions (yellow dashed lines) and π–π stacking inter­actions (black dashed lines).

Both helical chains in the structure have the same pitch length [10.8437 (5) Å], propagate along the *b*-axial direction and are alternately arranged *via* Ag1⋯Ag2 inter­actions [3.0814 (5) Å], resulting in the formation of a two-dimensional supra­molecular network extending parallel to the *ab* plane (Fig. 2 ▶). Furthermore, π–π stacking inter­actions [centroid–centroid distances = 3.514 (3) and 3.487 (3) Å] between pyridine rings of both helical chains contribute to the stabilization of the two-dimensional network. In the crystal structure, the two-dimensional networks are further stabilized by Ag⋯O and Ag⋯F inter­actions [Ag1⋯O1 2.861 (4), Ag1⋯O4 2.624 (4), Ag2⋯O2 2.617 (3), Ag2⋯F3^iv^ 3.017 (3) Å; symmetry code: (iv) −*x*, *y* − 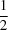, −*z* + 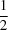] (Figs. 1 ▶ and 2 ▶) as well as N—H⋯O and N—H⋯O and C—H⋯O and C—H⋯F hydrogen-bonds (Table 1 ▶) between the helical chains and CF_3_SO_3_^−^ anions.

## Synthesis and crystallization   

The ligand (*N*-(pyridin-2-ylmeth­yl)pyridine-3-amine) was prepared according to a procedure described by Lee *et al.* (2013 ▶). Crystals of the title compound suitable for X-ray analysis were obtained by vapour diffusion of diethyl ether into a DMSO solution of the white precipitate afforded by the reaction of the ligand with silver(I) hexa­fluorido­phosphate in the molar ratio 1:1 in methanol.

## Refinement details   

Crystal data, data collection and structure refinement details are summarized in Table 2 ▶. All H atoms were positioned geometrically and refined using a riding model, with *d*(C—H) = 0.95 Å for C*sp* ^2^—H, 0.88 Å for amine N—H and 0.99 Å for methyl­ene C—H. For all H atoms *U*_iso_(H) = 1.2*U*_eq_(C,N).

## Supplementary Material

Crystal structure: contains datablock(s) I, New_Global_Publ_Block. DOI: 10.1107/S1600536814022922/zs2318sup1.cif

Structure factors: contains datablock(s) I. DOI: 10.1107/S1600536814022922/zs2318Isup2.hkl

CCDC reference: 1029928

Additional supporting information:  crystallographic information; 3D view; checkCIF report

## Figures and Tables

**Table 1:** Hydrogen-bond geometry (, )

*D*H*A*	*D*H	H*A*	*D* *A*	*D*H*A*
N3H3O6^i^	0.88	2.51	3.206(6)	136
N6H6O3^ii^	0.88	2.43	3.217(6)	149
C1H1O5^iii^	0.95	2.55	3.339(6)	141
C4H4O3^ii^	0.95	2.54	3.383(6)	147
C6H6*A*O5^i^	0.99	2.57	3.471(6)	151
C11H11F3^iii^	0.95	2.49	3.311(6)	145
C11H11O1^iii^	0.95	2.54	3.350(7)	143
C15H15O6^ii^	0.95	2.53	3.450(6)	164
C16H16O6^iv^	0.95	2.58	3.316(6)	135
C17H17*B*O3	0.99	2.58	3.422(6)	143

Symmetry codes: (i) 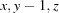; (ii) 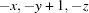; (iii) 
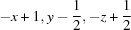
; (iv) 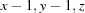.

**Table 2:** Experimental details

Crystal data	
Chemical formula	[Ag(C_11_H_11_N_3_)]CF_3_SO_3_
*M*_r_	442.17
Crystal system, space group	Monoclinic, *P*2_1_/*c*
Temperature (K)	173
*a*, *b*, *c* ()	13.7529(6), 10.8437(5), 19.5795(9)
()	99.826(1)
*V* (^3^)	2877.1(2)
*Z*	8
Radiation type	Mo *K*
(mm^1^)	1.60
Crystal size (mm)	0.31 0.22 0.10

Data collection	
Diffractometer	Bruker *SMART* CCD area detector
Absorption correction	Multi-scan (*SADABS*; Bruker, 2000 ▶)
*T*_min_, *T*_max_	0.637, 0.857
No. of measured, independent and observed [*I* > 2(*I*)] reflections	15852, 5629, 4286
*R*_int_	0.038
(sin /)_max_ (^1^)	0.617

Refinement	
*R*[*F* ^2^ > 2(*F* ^2^)], *wR*(*F* ^2^), *S*	0.039, 0.093, 1.06
No. of reflections	5629
No. of parameters	415
H-atom treatment	H-atom parameters constrained
_max_, _min_ (e ^3^)	1.04, 0.67

Computer programs: *SMART* and *SAINT-Plus* (Bruker, 2000 ▶), *SHELXS97*, *SHELXL97* and *SHELXTL* (Sheldrick, 2008 ▶) and *DIAMOND* (Brandenburg, 2005 ▶).

## supplementary crystallographic information

### Crystal data

**Table:**

[Ag(C_11_H_11_N_3_)]·CF_3_SO_3_	*F*(000) = 1744
*M**_r_* = 442.17	*D*_x_ = 2.042 Mg m^−^^3^
Monoclinic, *P*2_1_/*c*	Mo *K*α radiation, λ = 0.71073 Å
Hall symbol: -P 2ybc	Cell parameters from 5639 reflections
*a* = 13.7529 (6) Å	θ = 2.4–28.2°
*b* = 10.8437 (5) Å	µ = 1.60 mm^−^^1^
*c* = 19.5795 (9) Å	*T* = 173 K
β = 99.826 (1)°	Plate, colorless
*V* = 2877.1 (2) Å^3^	0.31 × 0.22 × 0.10 mm
*Z* = 8	

### Data collection

**Table:**

Bruker SMART CCD area detector diffractometer	5629 independent reflections
Radiation source: fine-focus sealed tube	4286 reflections with *I* > 2σ(*I*)
Graphite monochromator	*R*_int_ = 0.038
φ and ω scans	θ_max_ = 26.0°, θ_min_ = 1.5°
Absorption correction: multi-scan (*SADABS*; Bruker, 2000)	*h* = −16→16
*T*_min_ = 0.637, *T*_max_ = 0.857	*k* = −13→9
15852 measured reflections	*l* = −23→24

### Refinement

**Table:**

Refinement on *F*^2^	Primary atom site location: structure-invariant direct methods
Least-squares matrix: full	Secondary atom site location: difference Fourier map
*R*[*F*^2^ > 2σ(*F*^2^)] = 0.039	Hydrogen site location: inferred from neighbouring sites
*wR*(*F*^2^) = 0.093	H-atom parameters constrained
*S* = 1.06	*w* = 1/[σ^2^(*F*_o_^2^) + (0.0369*P*)^2^ + 6.3774*P*] where *P* = (*F*_o_^2^ + 2*F*_c_^2^)/3
5629 reflections	(Δ/σ)_max_ = 0.001
415 parameters	Δρ_max_ = 1.04 e Å^−^^3^
0 restraints	Δρ_min_ = −0.67 e Å^−^^3^

### Special details

**Table:**

Geometry. All e.s.d.'s (except the e.s.d. in the dihedral angle between two l.s. planes) are estimated using the full covariance matrix. The cell e.s.d.'s are taken into account individually in the estimation of e.s.d.'s in distances, angles and torsion angles; correlations between e.s.d.'s in cell parameters are only used when they are defined by crystal symmetry. An approximate (isotropic) treatment of cell e.s.d.'s is used for estimating e.s.d.'s involving l.s. planes.
Refinement. Refinement of *F*^2^ against ALL reflections. The weighted *R*-factor *wR* and goodness of fit *S* are based on *F*^2^, conventional *R*-factors *R* are based on *F*, with *F* set to zero for negative *F*^2^. The threshold expression of *F*^2^ > σ(*F*^2^) is used only for calculating *R*-factors(gt) *etc*. and is not relevant to the choice of reflections for refinement. *R*-factors based on *F*^2^ are statistically about twice as large as those based on *F*, and *R*- factors based on ALL data will be even larger.

### Fractional atomic coordinates and isotropic or equivalent isotropic displacement parameters (Å^2^)

**Table:**

	*x*	*y*	*z*	*U*_iso_*/*U*_eq_	
Ag1	0.30869 (3)	0.63691 (3)	0.192614 (19)	0.02491 (11)	
Ag2	0.17632 (3)	0.43820 (3)	0.24204 (2)	0.02787 (11)	
N1	0.3183 (3)	0.4818 (4)	0.1251 (2)	0.0220 (9)	
N2	0.7006 (3)	0.2705 (3)	0.2265 (2)	0.0223 (9)	
N3	0.4684 (3)	0.2087 (4)	0.1066 (2)	0.0284 (10)	
H3	0.4727	0.1538	0.0742	0.034*	
C1	0.3913 (3)	0.3969 (4)	0.1368 (2)	0.0231 (11)	
H1	0.4428	0.4078	0.1753	0.028*	
C2	0.3940 (3)	0.2938 (4)	0.0943 (2)	0.0228 (10)	
C3	0.3168 (3)	0.2794 (5)	0.0383 (2)	0.0252 (11)	
H3A	0.3152	0.2103	0.0083	0.030*	
C4	0.2434 (4)	0.3662 (5)	0.0272 (3)	0.0275 (11)	
H4	0.1904	0.3569	−0.0104	0.033*	
C5	0.2462 (4)	0.4662 (5)	0.0701 (2)	0.0237 (11)	
H5	0.1956	0.5265	0.0607	0.028*	
C6	0.5398 (4)	0.2033 (5)	0.1695 (3)	0.0288 (12)	
H6A	0.5618	0.1167	0.1774	0.035*	
H6B	0.5071	0.2279	0.2087	0.035*	
C7	0.6299 (4)	0.2836 (4)	0.1702 (3)	0.0234 (11)	
C8	0.6403 (4)	0.3658 (5)	0.1181 (3)	0.0327 (12)	
H8	0.5897	0.3735	0.0787	0.039*	
C9	0.7247 (4)	0.4367 (5)	0.1237 (3)	0.0346 (13)	
H9	0.7330	0.4928	0.0879	0.042*	
C10	0.7964 (4)	0.4258 (5)	0.1812 (3)	0.0324 (12)	
H10	0.8543	0.4751	0.1868	0.039*	
C11	0.7815 (4)	0.3405 (5)	0.2307 (3)	0.0314 (12)	
H11	0.8318	0.3310	0.2701	0.038*	
N4	−0.1913 (3)	0.0415 (3)	0.1616 (2)	0.0218 (9)	
N5	0.1834 (3)	0.2634 (4)	0.1887 (2)	0.0224 (9)	
N6	−0.0542 (3)	0.2536 (4)	0.0675 (2)	0.0295 (10)	
H6	−0.0559	0.2757	0.0241	0.035*	
C12	−0.1212 (3)	0.1180 (4)	0.1464 (2)	0.0211 (10)	
H12	−0.0651	0.1334	0.1810	0.025*	
C13	−0.1267 (3)	0.1755 (4)	0.0824 (2)	0.0221 (10)	
C14	−0.2106 (4)	0.1521 (4)	0.0325 (2)	0.0273 (11)	
H14	−0.2181	0.1902	−0.0118	0.033*	
C15	−0.2815 (4)	0.0739 (5)	0.0483 (3)	0.0306 (12)	
H15	−0.3385	0.0570	0.0148	0.037*	
C16	−0.2702 (4)	0.0194 (4)	0.1130 (3)	0.0261 (11)	
H16	−0.3197	−0.0352	0.1233	0.031*	
C17	0.0244 (3)	0.3012 (5)	0.1190 (3)	0.0271 (11)	
H17A	−0.0031	0.3203	0.1614	0.033*	
H17B	0.0475	0.3798	0.1016	0.033*	
C18	0.1121 (4)	0.2185 (4)	0.1389 (2)	0.0227 (10)	
C19	0.1216 (4)	0.1044 (5)	0.1089 (3)	0.0313 (12)	
H19	0.0706	0.0738	0.0742	0.038*	
C20	0.2063 (4)	0.0352 (5)	0.1301 (3)	0.0351 (13)	
H20	0.2139	−0.0430	0.1098	0.042*	
C21	0.2788 (4)	0.0807 (5)	0.1806 (3)	0.0340 (13)	
H21	0.3372	0.0348	0.1961	0.041*	
C22	0.2647 (4)	0.1953 (5)	0.2082 (3)	0.0281 (11)	
H22	0.3152	0.2273	0.2428	0.034*	
S1	0.03658 (9)	0.65963 (11)	0.12069 (6)	0.0230 (3)	
O1	0.1266 (3)	0.7218 (3)	0.1137 (2)	0.0390 (9)	
O2	0.0406 (3)	0.5931 (3)	0.18476 (18)	0.0343 (9)	
O3	−0.0106 (3)	0.5974 (3)	0.05959 (18)	0.0369 (9)	
C23	−0.0469 (4)	0.7861 (5)	0.1303 (3)	0.0289 (12)	
F1	−0.0609 (3)	0.8570 (3)	0.07437 (17)	0.0480 (9)	
F2	−0.1350 (2)	0.7440 (3)	0.13945 (17)	0.0439 (8)	
F3	−0.0113 (2)	0.8558 (3)	0.18486 (16)	0.0381 (8)	
S2	0.47697 (9)	0.84803 (12)	0.12529 (6)	0.0269 (3)	
O4	0.3758 (3)	0.8093 (3)	0.1188 (2)	0.0380 (9)	
O5	0.5228 (3)	0.8873 (3)	0.19320 (19)	0.0403 (10)	
O6	0.4983 (3)	0.9245 (4)	0.07004 (19)	0.0410 (10)	
C24	0.5413 (4)	0.7040 (5)	0.1142 (3)	0.0311 (12)	
F4	0.5337 (2)	0.6255 (3)	0.16634 (16)	0.0347 (7)	
F5	0.6375 (2)	0.7235 (3)	0.11623 (17)	0.0428 (8)	
F6	0.5059 (3)	0.6481 (3)	0.05552 (17)	0.0539 (10)	

### Atomic displacement parameters (Å^2^)

**Table:**

	*U*^11^	*U*^22^	*U*^33^	*U*^12^	*U*^13^	*U*^23^
Ag1	0.0246 (2)	0.0220 (2)	0.0286 (2)	−0.00393 (16)	0.00586 (15)	−0.00514 (16)
Ag2	0.0321 (2)	0.0252 (2)	0.0282 (2)	−0.00139 (17)	0.01060 (17)	−0.00680 (17)
N1	0.020 (2)	0.021 (2)	0.025 (2)	−0.0021 (17)	0.0048 (17)	0.0009 (17)
N2	0.022 (2)	0.016 (2)	0.029 (2)	0.0018 (16)	0.0050 (17)	−0.0032 (17)
N3	0.033 (2)	0.020 (2)	0.031 (2)	0.0039 (18)	0.0014 (19)	−0.0082 (18)
C1	0.022 (3)	0.027 (3)	0.021 (2)	−0.005 (2)	0.002 (2)	−0.001 (2)
C2	0.027 (3)	0.019 (2)	0.023 (3)	−0.002 (2)	0.006 (2)	0.001 (2)
C3	0.029 (3)	0.025 (3)	0.022 (3)	−0.005 (2)	0.005 (2)	−0.004 (2)
C4	0.025 (3)	0.032 (3)	0.024 (3)	−0.004 (2)	−0.001 (2)	0.002 (2)
C5	0.022 (3)	0.027 (3)	0.022 (3)	0.000 (2)	0.004 (2)	0.003 (2)
C6	0.024 (3)	0.024 (3)	0.038 (3)	0.004 (2)	0.007 (2)	0.002 (2)
C7	0.027 (3)	0.015 (2)	0.029 (3)	0.003 (2)	0.006 (2)	−0.004 (2)
C8	0.040 (3)	0.030 (3)	0.029 (3)	0.003 (2)	0.005 (2)	0.001 (2)
C9	0.046 (3)	0.023 (3)	0.038 (3)	0.004 (2)	0.018 (3)	0.009 (2)
C10	0.032 (3)	0.021 (3)	0.049 (3)	−0.002 (2)	0.018 (3)	−0.001 (2)
C11	0.024 (3)	0.029 (3)	0.040 (3)	0.001 (2)	0.002 (2)	−0.004 (2)
N4	0.025 (2)	0.016 (2)	0.025 (2)	0.0020 (16)	0.0047 (17)	−0.0005 (17)
N5	0.029 (2)	0.019 (2)	0.021 (2)	0.0012 (17)	0.0104 (17)	0.0035 (17)
N6	0.035 (2)	0.033 (2)	0.020 (2)	−0.006 (2)	0.0030 (18)	0.0102 (19)
C12	0.021 (2)	0.020 (2)	0.021 (2)	−0.0001 (19)	0.001 (2)	−0.0012 (19)
C13	0.026 (3)	0.016 (2)	0.025 (3)	0.0041 (19)	0.004 (2)	0.000 (2)
C14	0.038 (3)	0.023 (3)	0.017 (2)	0.005 (2)	−0.003 (2)	0.000 (2)
C15	0.027 (3)	0.028 (3)	0.033 (3)	0.000 (2)	−0.007 (2)	−0.009 (2)
C16	0.022 (3)	0.020 (3)	0.036 (3)	0.001 (2)	0.006 (2)	−0.001 (2)
C17	0.026 (3)	0.024 (3)	0.031 (3)	−0.003 (2)	0.005 (2)	0.003 (2)
C18	0.029 (3)	0.018 (2)	0.025 (3)	−0.003 (2)	0.013 (2)	0.001 (2)
C19	0.039 (3)	0.024 (3)	0.032 (3)	−0.006 (2)	0.011 (2)	−0.001 (2)
C20	0.051 (4)	0.023 (3)	0.035 (3)	0.002 (3)	0.019 (3)	−0.004 (2)
C21	0.035 (3)	0.031 (3)	0.038 (3)	0.004 (2)	0.011 (3)	0.004 (3)
C22	0.030 (3)	0.027 (3)	0.029 (3)	0.000 (2)	0.011 (2)	0.004 (2)
S1	0.0249 (6)	0.0222 (6)	0.0208 (6)	0.0063 (5)	0.0006 (5)	−0.0015 (5)
O1	0.029 (2)	0.037 (2)	0.054 (2)	0.0034 (17)	0.0158 (18)	−0.0033 (19)
O2	0.046 (2)	0.031 (2)	0.0251 (19)	0.0098 (17)	0.0012 (17)	0.0060 (16)
O3	0.044 (2)	0.033 (2)	0.030 (2)	0.0092 (17)	−0.0026 (17)	−0.0112 (17)
C23	0.029 (3)	0.028 (3)	0.028 (3)	0.004 (2)	−0.001 (2)	−0.006 (2)
F1	0.062 (2)	0.0397 (19)	0.0404 (19)	0.0274 (17)	0.0022 (16)	0.0099 (16)
F2	0.0218 (16)	0.052 (2)	0.058 (2)	0.0028 (15)	0.0059 (15)	−0.0139 (17)
F3	0.0315 (16)	0.0381 (18)	0.0420 (18)	0.0053 (14)	−0.0013 (14)	−0.0205 (15)
S2	0.0274 (7)	0.0256 (7)	0.0266 (7)	−0.0033 (5)	0.0018 (5)	0.0070 (5)
O4	0.030 (2)	0.031 (2)	0.055 (3)	0.0000 (16)	0.0120 (18)	0.0148 (19)
O5	0.061 (3)	0.028 (2)	0.030 (2)	−0.0019 (19)	0.0017 (19)	−0.0031 (17)
O6	0.035 (2)	0.048 (2)	0.036 (2)	−0.0173 (18)	−0.0061 (17)	0.0191 (19)
C24	0.031 (3)	0.038 (3)	0.027 (3)	−0.005 (2)	0.011 (2)	−0.005 (2)
F4	0.0365 (17)	0.0227 (15)	0.0456 (18)	0.0006 (13)	0.0087 (14)	0.0061 (14)
F5	0.0282 (17)	0.049 (2)	0.055 (2)	−0.0043 (15)	0.0179 (15)	−0.0013 (17)
F6	0.061 (2)	0.061 (2)	0.0378 (19)	−0.0073 (19)	0.0040 (17)	−0.0228 (18)

### Geometric parameters (Å, º)

**Table:**

Ag1—N1	2.156 (4)	N6—C13	1.377 (6)
Ag1—N2^i^	2.167 (4)	N6—C17	1.443 (6)
Ag1—Ag2	3.0814 (5)	N6—H6	0.8800
Ag2—N4^ii^	2.174 (4)	C12—C13	1.390 (7)
Ag2—N5	2.175 (4)	C12—H12	0.9500
N1—C5	1.344 (6)	C13—C14	1.402 (7)
N1—C1	1.353 (6)	C14—C15	1.367 (7)
N2—C11	1.338 (6)	C14—H14	0.9500
N2—C7	1.347 (6)	C15—C16	1.383 (7)
N2—Ag1^iii^	2.167 (4)	C15—H15	0.9500
N3—C2	1.369 (6)	C16—H16	0.9500
N3—C6	1.439 (6)	C17—C18	1.500 (7)
N3—H3	0.8800	C17—H17A	0.9900
C1—C2	1.398 (7)	C17—H17B	0.9900
C1—H1	0.9500	C18—C19	1.385 (7)
C2—C3	1.398 (6)	C19—C20	1.389 (8)
C3—C4	1.369 (7)	C19—H19	0.9500
C3—H3A	0.9500	C20—C21	1.370 (8)
C4—C5	1.368 (7)	C20—H20	0.9500
C4—H4	0.9500	C21—C22	1.381 (7)
C5—H5	0.9500	C21—H21	0.9500
C6—C7	1.512 (7)	C22—H22	0.9500
C6—H6A	0.9900	S1—O3	1.429 (4)
C6—H6B	0.9900	S1—O1	1.436 (4)
C7—C8	1.380 (7)	S1—O2	1.440 (4)
C8—C9	1.382 (8)	S1—C23	1.819 (5)
C8—H8	0.9500	C23—F1	1.324 (6)
C9—C10	1.369 (8)	C23—F3	1.332 (5)
C9—H9	0.9500	C23—F2	1.336 (6)
C10—C11	1.379 (7)	S2—O6	1.433 (4)
C10—H10	0.9500	S2—O5	1.435 (4)
C11—H11	0.9500	S2—O4	1.438 (4)
N4—C16	1.336 (6)	S2—C24	1.826 (6)
N4—C12	1.343 (6)	C24—F6	1.317 (6)
N4—Ag2^iv^	2.174 (4)	C24—F5	1.334 (6)
N5—C22	1.341 (6)	C24—F4	1.347 (6)
N5—C18	1.349 (6)		
			
N1—Ag1—N2^i^	170.69 (14)	N4—C12—C13	123.1 (4)
N1—Ag1—Ag2	75.41 (10)	N4—C12—H12	118.4
N2^i^—Ag1—Ag2	97.25 (10)	C13—C12—H12	118.4
N4^ii^—Ag2—N5	149.42 (14)	N6—C13—C12	122.5 (4)
N4^ii^—Ag2—Ag1	86.49 (10)	N6—C13—C14	120.2 (4)
N5—Ag2—Ag1	112.41 (10)	C12—C13—C14	117.3 (4)
C5—N1—C1	118.3 (4)	C15—C14—C13	119.3 (5)
C5—N1—Ag1	118.4 (3)	C15—C14—H14	120.4
C1—N1—Ag1	123.3 (3)	C13—C14—H14	120.4
C11—N2—C7	117.9 (4)	C14—C15—C16	120.0 (5)
C11—N2—Ag1^iii^	119.1 (3)	C14—C15—H15	120.0
C7—N2—Ag1^iii^	122.9 (3)	C16—C15—H15	120.0
C2—N3—C6	124.0 (4)	N4—C16—C15	121.7 (5)
C2—N3—H3	118.0	N4—C16—H16	119.1
C6—N3—H3	118.0	C15—C16—H16	119.1
N1—C1—C2	122.6 (4)	N6—C17—C18	116.2 (4)
N1—C1—H1	118.7	N6—C17—H17A	108.2
C2—C1—H1	118.7	C18—C17—H17A	108.2
N3—C2—C1	121.9 (4)	N6—C17—H17B	108.2
N3—C2—C3	120.5 (4)	C18—C17—H17B	108.2
C1—C2—C3	117.5 (4)	H17A—C17—H17B	107.4
C4—C3—C2	119.2 (5)	N5—C18—C19	121.3 (5)
C4—C3—H3A	120.4	N5—C18—C17	115.1 (4)
C2—C3—H3A	120.4	C19—C18—C17	123.6 (5)
C5—C4—C3	120.3 (5)	C18—C19—C20	119.4 (5)
C5—C4—H4	119.8	C18—C19—H19	120.3
C3—C4—H4	119.8	C20—C19—H19	120.3
N1—C5—C4	122.1 (5)	C21—C20—C19	119.4 (5)
N1—C5—H5	119.0	C21—C20—H20	120.3
C4—C5—H5	119.0	C19—C20—H20	120.3
N3—C6—C7	115.1 (4)	C20—C21—C22	118.3 (5)
N3—C6—H6A	108.5	C20—C21—H21	120.9
C7—C6—H6A	108.5	C22—C21—H21	120.9
N3—C6—H6B	108.5	N5—C22—C21	123.3 (5)
C7—C6—H6B	108.5	N5—C22—H22	118.3
H6A—C6—H6B	107.5	C21—C22—H22	118.3
N2—C7—C8	121.4 (5)	O3—S1—O1	114.7 (2)
N2—C7—C6	115.0 (4)	O3—S1—O2	115.9 (2)
C8—C7—C6	123.6 (5)	O1—S1—O2	114.3 (2)
C7—C8—C9	119.5 (5)	O3—S1—C23	103.8 (2)
C7—C8—H8	120.3	O1—S1—C23	103.0 (2)
C9—C8—H8	120.3	O2—S1—C23	102.7 (2)
C10—C9—C8	119.7 (5)	F1—C23—F3	108.4 (4)
C10—C9—H9	120.2	F1—C23—F2	107.6 (4)
C8—C9—H9	120.2	F3—C23—F2	107.6 (4)
C9—C10—C11	117.6 (5)	F1—C23—S1	111.0 (4)
C9—C10—H10	121.2	F3—C23—S1	111.0 (3)
C11—C10—H10	121.2	F2—C23—S1	111.0 (4)
N2—C11—C10	123.9 (5)	O6—S2—O5	114.4 (2)
N2—C11—H11	118.0	O6—S2—O4	115.0 (2)
C10—C11—H11	118.0	O5—S2—O4	115.7 (2)
C16—N4—C12	118.7 (4)	O6—S2—C24	103.9 (2)
C16—N4—Ag2^iv^	118.0 (3)	O5—S2—C24	102.6 (2)
C12—N4—Ag2^iv^	123.0 (3)	O4—S2—C24	102.7 (2)
C22—N5—C18	118.4 (4)	F6—C24—F5	108.4 (4)
C22—N5—Ag2	116.5 (3)	F6—C24—F4	107.8 (4)
C18—N5—Ag2	125.1 (3)	F5—C24—F4	106.4 (4)
C13—N6—C17	123.7 (4)	F6—C24—S2	112.5 (4)
C13—N6—H6	118.2	F5—C24—S2	111.0 (4)
C17—N6—H6	118.2	F4—C24—S2	110.4 (3)
			
N1—Ag1—Ag2—N4^ii^	160.70 (15)	N4—C12—C13—N6	−179.9 (4)
N2^i^—Ag1—Ag2—N4^ii^	−13.47 (14)	N4—C12—C13—C14	0.6 (7)
N1—Ag1—Ag2—N5	5.50 (15)	N6—C13—C14—C15	179.7 (5)
N2^i^—Ag1—Ag2—N5	−168.67 (15)	C12—C13—C14—C15	−0.8 (7)
Ag2—Ag1—N1—C5	82.5 (3)	C13—C14—C15—C16	0.3 (7)
Ag2—Ag1—N1—C1	−95.0 (4)	C12—N4—C16—C15	−0.6 (7)
C5—N1—C1—C2	−0.6 (7)	Ag2^iv^—N4—C16—C15	−174.9 (4)
Ag1—N1—C1—C2	177.0 (3)	C14—C15—C16—N4	0.4 (8)
C6—N3—C2—C1	11.5 (7)	C13—N6—C17—C18	83.2 (6)
C6—N3—C2—C3	−167.8 (5)	C22—N5—C18—C19	0.6 (7)
N1—C1—C2—N3	−180.0 (4)	Ag2—N5—C18—C19	−178.2 (3)
N1—C1—C2—C3	−0.6 (7)	C22—N5—C18—C17	−178.9 (4)
N3—C2—C3—C4	−180.0 (4)	Ag2—N5—C18—C17	2.3 (6)
C1—C2—C3—C4	0.6 (7)	N6—C17—C18—N5	−178.1 (4)
C2—C3—C4—C5	0.5 (7)	N6—C17—C18—C19	2.3 (7)
C1—N1—C5—C4	1.8 (7)	N5—C18—C19—C20	−0.4 (7)
Ag1—N1—C5—C4	−175.9 (4)	C17—C18—C19—C20	179.1 (5)
C3—C4—C5—N1	−1.8 (7)	C18—C19—C20—C21	0.3 (8)
C2—N3—C6—C7	−87.1 (6)	C19—C20—C21—C22	−0.4 (8)
C11—N2—C7—C8	0.5 (7)	C18—N5—C22—C21	−0.7 (7)
Ag1^iii^—N2—C7—C8	−177.0 (4)	Ag2—N5—C22—C21	178.1 (4)
C11—N2—C7—C6	−179.0 (4)	C20—C21—C22—N5	0.7 (8)
Ag1^iii^—N2—C7—C6	3.6 (6)	O3—S1—C23—F1	−57.7 (4)
N3—C6—C7—N2	−174.5 (4)	O1—S1—C23—F1	62.2 (4)
N3—C6—C7—C8	6.1 (7)	O2—S1—C23—F1	−178.8 (4)
N2—C7—C8—C9	−0.3 (8)	O3—S1—C23—F3	−178.4 (4)
C6—C7—C8—C9	179.1 (5)	O1—S1—C23—F3	−58.5 (4)
C7—C8—C9—C10	−0.8 (8)	O2—S1—C23—F3	60.5 (4)
C8—C9—C10—C11	1.7 (8)	O3—S1—C23—F2	61.9 (4)
C7—N2—C11—C10	0.5 (7)	O1—S1—C23—F2	−178.2 (3)
Ag1^iii^—N2—C11—C10	178.1 (4)	O2—S1—C23—F2	−59.2 (4)
C9—C10—C11—N2	−1.6 (8)	O6—S2—C24—F6	−64.1 (4)
N4^ii^—Ag2—N5—C22	−48.9 (5)	O5—S2—C24—F6	176.4 (4)
Ag1—Ag2—N5—C22	75.7 (3)	O4—S2—C24—F6	56.0 (4)
N4^ii^—Ag2—N5—C18	129.9 (4)	O6—S2—C24—F5	57.6 (4)
Ag1—Ag2—N5—C18	−105.5 (3)	O5—S2—C24—F5	−61.9 (4)
C16—N4—C12—C13	0.1 (7)	O4—S2—C24—F5	177.7 (3)
Ag2^iv^—N4—C12—C13	174.1 (3)	O6—S2—C24—F4	175.4 (3)
C17—N6—C13—C12	−11.8 (7)	O5—S2—C24—F4	56.0 (4)
C17—N6—C13—C14	167.7 (4)	O4—S2—C24—F4	−64.5 (4)

Symmetry codes: (i) −*x*+1, *y*+1/2, −*z*+1/2; (ii) −*x*, *y*+1/2, −*z*+1/2; (iii) −*x*+1, *y*−1/2, −*z*+1/2; (iv) −*x*, *y*−1/2, −*z*+1/2.

### Hydrogen-bond geometry (Å, º)

**Table:**

*D*—H···*A*	*D*—H	H···*A*	*D*···*A*	*D*—H···*A*
N3—H3···O6^v^	0.88	2.51	3.206 (6)	136
N6—H6···O3^vi^	0.88	2.43	3.217 (6)	149
C1—H1···O5^iii^	0.95	2.55	3.339 (6)	141
C4—H4···O3^vi^	0.95	2.54	3.383 (6)	147
C6—H6*A*···O5^v^	0.99	2.57	3.471 (6)	151
C8—H8···N3	0.95	2.57	2.891 (7)	100
C11—H11···F3^iii^	0.95	2.49	3.311 (6)	145
C11—H11···O1^iii^	0.95	2.54	3.350 (7)	143
C15—H15···O6^vi^	0.95	2.53	3.450 (6)	164
C16—H16···O6^vii^	0.95	2.58	3.316 (6)	135
C17—H17*B*···O3	0.99	2.58	3.422 (6)	143
C19—H19···N6	0.95	2.59	2.906 (7)	100

Symmetry codes: (iii) −*x*+1, *y*−1/2, −*z*+1/2; (v) *x*, *y*−1, *z*; (vi) −*x*, −*y*+1, −*z*; (vii) *x*−1, *y*−1, *z*.
